# The poly-omics of ageing through individual-based metabolic modelling

**DOI:** 10.1186/s12859-018-2383-z

**Published:** 2018-11-20

**Authors:** Elisabeth Yaneske, Claudio Angione

**Affiliations:** Department of Computer Science and Information Systems, Teesside University, Borough Road, Middlesbrough, UK

**Keywords:** Ageing, Biological age, Metabolic age, Metabolic modelling, Flux balance analysis, Poly-omics, Machine learning, CD4 T-cells

## Abstract

**Background:**

Ageing can be classified in two different ways, chronological ageing and biological ageing. While chronological age is a measure of the time that has passed since birth, biological (also known as transcriptomic) ageing is defined by how time and the environment affect an individual in comparison to other individuals of the same chronological age. Recent research studies have shown that transcriptomic age is associated with certain genes, and that each of those genes has an effect size. Using these effect sizes we can calculate the transcriptomic age of an individual from their age-associated gene expression levels. The limitation of this approach is that it does not consider how these changes in gene expression affect the metabolism of individuals and hence their observable cellular phenotype.

**Results:**

We propose a method based on poly-omic constraint-based models and machine learning in order to further the understanding of transcriptomic ageing. We use normalised CD4 T-cell gene expression data from peripheral blood mononuclear cells in 499 healthy individuals to create individual metabolic models. These models are then combined with a transcriptomic age predictor and chronological age to provide new insights into the differences between transcriptomic and chronological ageing. As a result, we propose a novel metabolic age predictor.

**Conclusions:**

We show that our poly-omic predictors provide a more detailed analysis of transcriptomic ageing compared to gene-based approaches, and represent a basis for furthering our knowledge of the ageing mechanisms in human cells.

**Electronic supplementary material:**

The online version of this article (10.1186/s12859-018-2383-z) contains supplementary material, which is available to authorized users.

## Background

Ageing is a complex process characterised by phenotypes such as greying hair and wrinkles, as well as age-associated diseases such as cancer, osteoarthritis and cardiovascular disease. Phenotypes of ageing and age-associated diseases can be linked to age-associated changes in metabolic subsystems [1–3]. Identifying these metabolic links has recently led to the discovery of age-associated biomarkers [4, 5].

There are many different theories of the underlying mechanisms of ageing, including the mitochondrial theory of ageing, accumulation of metabolic by-products and dysregulation of regulatory pathways. The mitochondrion is the primary organelle responsible for metabolic cellular respiration; it takes in oxygen and nutrients and converts them into energy in the form of adenosine triphosphate (ATP). The mitochondrial theory of ageing states that oxidative damage caused by reactive oxygen species (ROS) produced by the mitochondria contributes to ageing by causing damage to mitochondrial DNA, lipids and proteins, which ultimately leads to cell death [6, 7]. Mitochondrial dysfunction and oxidative damage have been linked to age-associated neurodegenerative disorders such as Alzheimer’s disease, Parkinson’s disease and Huntington’s disease [8, 9], as well as to the pathogenesis of cancer [10, 11].

Metabolism is increasingly being considered as a driver, rather than a marker, of the ageing process [12]. Three examples of metabolic by-products linked with ageing are amyloid proteins, advanced glycation end-products (AGEs) and lipofuscin. Accumulation of amyloid proteins in the central nervous system is associated with neurodegenerative disease in ageing [13, 14]; for instance, *β*-amyloid plaques in brain tissue are linked with the pathogenesis of Alzheimer’s disease [15, 16]. AGEs can be ingested in foods or formed in the body by non-enzymatic glycation of lipids, nucleic acids and proteins, and their accumulation is thought to contribute to the ageing process [17, 18]. AGEs are formed when foods are processed at high temperatures such as deep-frying, grilling and roasting. They can increase oxidative stress, upregulate inflammation, and form cross-links with proteins [19], which cause impaired elasticity to blood vessels, therefore leading to poor heart health [20, 21]. Upregulation of inflammation caused by AGEs has been also linked to cancer [22–24]. Eating raw foods or foods cooked at lower temperatures can help to reduce dietary intake of AGEs [17]. Lipofuscin is a non-degradable metabolic by-product that builds up in lysosomes with time, and has been associated with age-related cellular degeneration [25], particularly macular degeneration [26].

One important example of the dysregulation of regulatory pathways as we age is chronic inflammation. The term ’inflammaging’ was proposed by Franceschi et al. [27] to describe the imbalance between pro- and anti-inflammatory networks, which contributes to the chronic diseases of ageing. The function of the immune system declines as we age, leading to increased susceptibility to infectious diseases such as influenza [28], as well as decreased response to vaccinations against them [29]. This decline in function has been reported in CD4 T-cells [30, 31], which are used in this study, along with changes in the ageing transcriptome [32].

Age can be defined as chronological or transcriptomic/biological. Chronological age is a measure of the time that has passed since our birth, whereas transcriptomic age represents the difference in how time and the environment have affected the cells and organs of our body as compared to others of the same chronological age. Our transcriptomic age can therefore be older or younger than our chronological age. Until now, transcriptomic age has been calculated for individuals using transcriptomic-only data [33].

The limitation of this approach is that it does not take into account how age-associated gene expression affects the metabolism within cells and thus their observable cellular phenotype. This paper aims to improve on the current understanding of ageing (based on transcriptomics data alone) by modelling how age-associated gene expression changes metabolic processes, therefore enabling the identification of metabolic age predictors, selected using machine learning techniques.

Metabolic models have proven to be valuable computational tools to study metabolism, as they allow predicting phenotypes from genotypes. By modelling most of the known biochemistry of a cell, they allow achieving a mechanistic understanding of the genotype-phenotype relationship. Coupled with tools for integration of omics data, metabolic models have been successfully exploited in a wide range of applications in health and disease, including personalised, condition- and tissue-specific cancer modelling [34–37].

Gene expression data and other types of omics-derived data can be used to constrain metabolic models for phenotype prediction [38]. The process of linking metabolic networks to phenotypes enables a better prediction of cellular phenotype compared to predictions from gene expression alone [39]. Exploiting this idea to generate a poly-omic model of ageing, we first generate individual-based genome-scale metabolic models and the associated fluxomic profiles. Specifically, we use CD4 T-cell transcriptomics data to modify a constraint-based metabolic model and achieve the predicted flux distributions (fluxomic profiles) for each individual in the cohort. Then, we adapt machine learning techniques in order to investigate metabolic changes linked to the chronological age of the individuals. We compare transcriptomic- and fluxomic-based clustering with chronological age and find that metabolic models are a better predictor of chronological age [40].

Our poly-omic pipeline also enables us to identify metabolic biomarkers of ageing, which are validated by recent literature, and to obtain metabolic age predictors. As a result, we build a metabolic age predictor capable of calculating the metabolic ages of individuals. Although a small number of metabolomics biomarkers have been proposed [41], to our knowledge this is the first time genome-scale predictors have been identified. We conclude that moving towards a poly-omic understanding of biological ageing can help provide a more accurate prediction of biological age, therefore leading to more targeted therapies for ageing individuals in a variety of environmental and physiological conditions.

## Methods

### The poly-omic ageing pipeline

Our pipeline starts from a meta-analysis of CD4 T-cell data containing the gene expression levels from human peripheral blood mononuclear cells and the chronological ages of 499 healthy individuals in the Boston area, comprised of 294 females and 205 males [42]. As the CD4 T-cell expression data was profiled on Affymetrix Human Gene 1.0 ST microarrays, it was first normalised using RMA (Robust Multi-array Average) [43]. In absence of a control profile, the gene expression values were then divided by the mean value for their associated probe. Using this normalised gene expression data, the transcriptomic ages for all 499 individuals were calculated as described in the next subsection (formulas (1) and (2)).

**Fig. 1:**
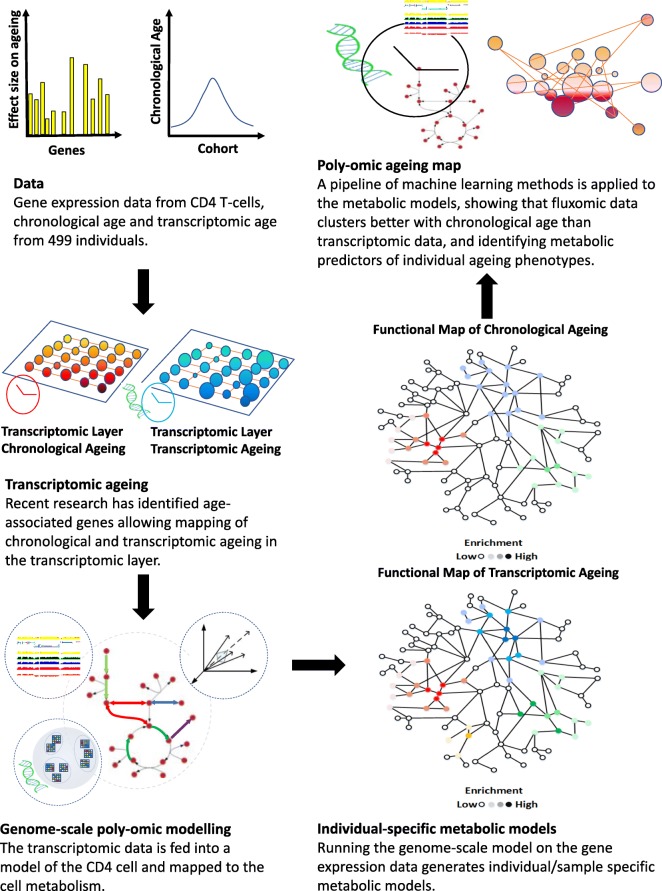
**Poly-omic ageing pipeline**. We start with the transcriptomic data and chronological ages from the CD4 T-cells of 499 individuals. We use the chronological data and corresponding age-associated transcriptomic predictors to obtain the effect of both chronological and transcriptomic ageing on the transcriptomic layer. We then combine with the functional biological network data determined by the metabolism and poly-omic model to obtain individual-based metabolic models and their fluxomic profiles. Finally, we adapt machine learning techniques to show that fluxomic data clusters better with chronological age than transcriptomic data, and to identify metabolic predictors of ageing (the poly-omic ageing map). Definition of key terms. **Transcriptomic** – gene expression data represented by a measurement of the mRNA transcripts within a cell. **Fluxomic** – fluxomic data refers to reaction flux rates, namely the value for the rate of metabolite conversion, measured in millimoles per hour per grams of dry weight, for each reaction or collection of related reactions (subsystems) within a cell. **Poly-omic** – the integration of more than one type of ’omic’ data e.g. transcriptomic and fluxomic data

Having obtained the transcriptomic ages, we then used individuals’ transcriptomic data to generate their personalised CD4 T-cell metabolic models. These were created using constraint based modelling of the CD4 T-cell [44] augmented with transcriptomics through GEMsplice [45], by setting individual constraints on the CD4 model (see the following subsections on constraint-based modelling for details on how the mapping was achieved). On the personalised models, we finally adapted a set of statistical and machine learning methods based on clustering, PCA analysis and elastic net regression to identify metabolic predictors of ageing. Our pipeline thus enabled us to progress to a poly-omic understanding of ageing in human cells (Fig. 1; see also the following subsections for details on the modelling approach adopted in this manuscript).

### Transcriptomic age predictor

The transcriptomic age of an individual within a sample can be calculated by first obtaining their transcriptomic predictor, *Z*, using the gene expression levels of 1497 age-associated genes (i.e. those found to be differentially expressed with chronological age). This is achieved through a linear combination of the expression levels, where coefficients are their associated effect sizes [33]:

1 $$ Z = \sum\limits_{i} b_{i} x_{i},   $$

where *x*_*i*_ is the gene expression level of the *i*th probe, and *b*_*i*_ is the effect size for the *i*th probe. Effect sizes were associated with individual genes, whereas the original data contained gene expression data associated with probes. Therefore, where a probe was mapped to more than one age-associated gene, the effect sizes for those genes were averaged to give an overall average effect size *b*_*i*_ for that probe.

The transcriptomic predictor for each individual is then scaled using the mean and standard deviation of the chronological ages, and the mean and standard deviation of the transcriptomic predictors from all the individuals in the sample [33]. This allows defining the *transcriptomic age* of an individual:

2 $$ \ SZ = \mu_{age} + \left(Z-\mu_{Z}\right) \frac{\sigma_{age}}{\sigma_{Z}},   $$

where *μ*_*age*_ and *σ*_*age*_ are the mean and the standard deviation of the chronological age across all the individuals within the sample, while *μ*_*Z*_ and *σ*_*Z*_ are the mean and the standard deviation of the predictor *Z* across all the individuals in the sample.

### Constraint-based modelling to generate individual-based metabolic models

Metabolic models can be analysed using constraint-based modelling and flux balance analysis (FBA), the most widely-used technique to simulate metabolic models at steady state [46]), to enable predictions of the distribution of reaction flux rates in the cell. Given the matrix *S* of all known metabolic biochemical reactions and their stoichiometry, and given the vector *v* of reaction flux rates in a given growth or physiological condition, the steady-state condition is set by the constraint *Sv*=0. Additional constraints are added on lower and upper bounds of *v* (*v*^*min*^ and *v*^*max*^). Constraints are included according to the growth or physiological condition that is simulated; these can also be set taking into account multiple omics data (e.g. transcriptomics data as used in our pipeline) [47]. Further constraints can include codon usage [48], splice-isoforms [45, 49], and can be analysed using pathway-oriented approaches [50, 51]. The metabolic network is then solved by maximising one or more cellular objectives (usually the biomass and energy-related or application-specific production of metabolites). For a comprehensive introduction to constraint-based metabolic modelling and its poly-omic extensions, the reader is referred to the reviews by Palsson and Vijayakumar et al. [52, 53].

As omics data to constrain the model, here we use transcriptomic data from each individual to generate personalised metabolic models. Through GEMsplice [45], we modify the upper- and lower- limits of reactions as a function of the expression levels of the genes involved in the reaction. More specifically, for each individual, to predict the cellular flux distribution (fluxomic profile) when multiple objectives have to be taken into account, we use the following bilevel linear program:

3 $$ \begin{aligned} & \text{max}\; g^{\intercal} v \\ & {\text{such that}} & \text{max}\;\; f^{\intercal} v, \quad Sv = 0,\\ & & v^{\text{min}} \varphi(\Theta) \leq v \leq v^{\text{max}} \varphi(\Theta). \end{aligned}   $$

The vectors *f* and *g* are weights to select (or combine) the objectives to be maximised from the vector *v*. The vector *Θ* represents the expression of a biochemical reaction, defined from the individual-based expression levels of its genes with a rule involving the max and min operators, depending on the type of enzyme (single gene, isozyme, or enzymatic complex). The function *φ*, which acts on *Θ*, converts the reaction expression values into coefficients for the bounds of reactions activated by those genes [54]. Here we set the primary objective *f* as biomass and the secondary objective *g* as ATP maintenance. Simulations were performed in Matlab.

### Cluster analysis

Cluster analysis was used in order to group individual response according to both their transcriptomic and fluxomic profiles, and visualise them with chronological age. We compared both agglomerative hierarchical clustering (AHC) and k-means clustering using a novel application of the silhouette method. The silhouette method calculates a value which is a measure of the similarity of the values within a cluster (cohesion) and the dissimilarity of the values within that cluster to other clusters (separation). The silhouette calculation gives a value between −1 and 1. Silhouette values close to 1 are desirable as they indicate a cluster has high cohesion and high separation; if most values are close to 1 then the number of clusters is a good representation of the data.

Here we use the silhouette value to measure the cohesion and separation of the clustering of individuals by chronological age [55]. We define the silhouette value of an individual within a cluster as:

4 $$ \ s(c) = \frac{l(c)-a(c)}{max(a(c),l(c))},   $$

where *s* is the silhouette value (−1≤*s*(*c*)≤1), *c* is the chronological age of the individual, *a* is the average dissimilarity of *c* to the other ages in the same cluster and *l* is the lowest average dissimilarity of *c* to any other age in a different cluster.

Our motivation for using the silhouette method was twofold. Firstly, we wanted to statistically compare the silhouette values for AHC and k-means to see which method performed better at clustering the data with chronological age. Secondly, we wanted to statistically compare whether transcriptomic-based or fluxomic-based clusters of individuals were consistent with chronological age.

### Principal component analysis

Multidimensional data such as fluxomic datasets can be visualised using Principal Component Analysis (PCA). PCA can reduce multidimensional datasets to as few as two or three latent dimensions (components), which allows inference of variables causing the largest variations in the data. Here we use PCA to identify the fluxes accounting for the greatest variation between individuals in different age groups according to chronological age. In our PCA analysis, the fluxomic data was split according to three chronological age groups: 21 and under (112 individuals: 64 female and 48 male), 22 to 49 (360 individuals: 219 female and 141 male), and 50 and over (27 individuals: 11 female and 16 male). The analysis was performed in R and visualised using FactoMineR [56].

The CD4 T-cell model contains 4229 flux variables (metabolic reactions). PCA analysis gives the contribution of each variable (reaction) to the variability of each component. The proportion of variability accounted for by a component is defined numerically by its eigenvalue. The total contribution *T*_*v*_ of a given variable across the components can be calculated by determining its overall weighted sum as follows:

5 $$ T_{v} = \sum\limits_{i=1}^{n} E_{i} V_{i},   $$

where *E*_*i*_ is the eigenvalue for the principal component *i*, *V*_*i*_ is the variable (reaction) contribution to the principal component *i*, and *n* is the number of components chosen to represent the data.

Variables (reactions) can be mapped to a subsystem (metabolic pathway), which contains a number of reactions that are interlinked to perform a cellular metabolic function. The CD4 T-cell model contains 95 pathways, each of which corresponds to a number of reactions and their flux values. A flux value for each pathway was calculated as the mean of its reaction flux values, and PCA was also performed on the pathway flux rates obtained for each individual. Similarly, the total contribution *T*_*s*_ of a given pathway across the components can be found using:

6 $$ T_{s} = \sum\limits_{i=1}^{n} E_{i} S_{i},   $$

where *E*_*i*_ is the eigenvalue for the principal component *i*, *S*_*i*_ is the subsystem (pathway) contribution to the principal component *i*, and *n* is the number of components.

### Elastic net regression

We use elastic net regression to identify metabolic predictors of chronological age and their effect sizes. Elastic net regression is a linear hybrid of the *L*_2_ penalty of ridge regression [57] and the *L*_1_ penalty of lasso regression [58]. For *α* between 0 and 1, where 0 is ridge regression and 1 is lasso regression, and a strictly non-negative *λ*, elastic net is defined as [59]:

7 $$ \left(\hat{\beta},\hat{\beta_{0}}\right) \,=\, \underset{\beta,\beta_{0}}{\text{argmin}} \left(\frac{1}{2N} \sum\limits_{i=1}^{N}\left(y_{i} - \beta_{0} - x^{T}_{i}\beta\right)^{2} \,+\, \lambda P_{\alpha}(\beta)\right),   $$

where

8 $$ \begin{aligned} P_{\alpha}(\beta) &= \frac{(1 - \alpha)}{2}\left\|\beta\right\|_{2}^{2} + \alpha \left\|\beta\right\|_{1} \\ &= {\sum\nolimits}_{j=1}^{p} \left(\frac{(1 - \alpha)}{2}\beta_{j}^{2} + \alpha \left|\beta_{j}\right|\right),  \end{aligned}  $$

*N* is the number of individuals, *y*_*i*_ is the chronological age of individual *i*, *x*_*i*_ is a *p*×1 vector of *p* metabolic pathway fluxes at individual *i*, *α* is set to 0.5 to achieve a balance between *L*_1_ and *L*_2_ norms, *λ* is a positive regularization parameter, *β*_0_ is a scalar parameter, and *β* is a *p*×1 vector of effect sizes on chronological age (regression coefficients), where *p* is the number of metabolic pathways. We also performed an equivalent analysis directly on reaction fluxes (Additional file 1).

Elastic net overcomes some of the limitations of using the lasso method alone [59]. When analysing high dimensional data, such as the individual by reaction data (499×4229), where the number of predictors is greater than the number of observations, the lasso method can only select at most the same number of variables as observations. However, omic data tends by its nature to often be highly correlated, which means there is high correlation between regression predictors. Where there is a group of highly correlated predictors, the lasso method will only select one variable from the group. The advantage of the elastic net method in our context is that while the *L*_1_ part of the penalty generates a sparse model, the quadratic part of the penalty (*L*_2_ regularisation ∥*β*∥^2^), taken from ridge regression, allows the number of selected variables to be greater than the number of observations, and allows groups of strongly correlated variables to be selected.

## Results and Discussion

### Clustering shows best dataset for prediction of chronological age

**Fig. 2:**
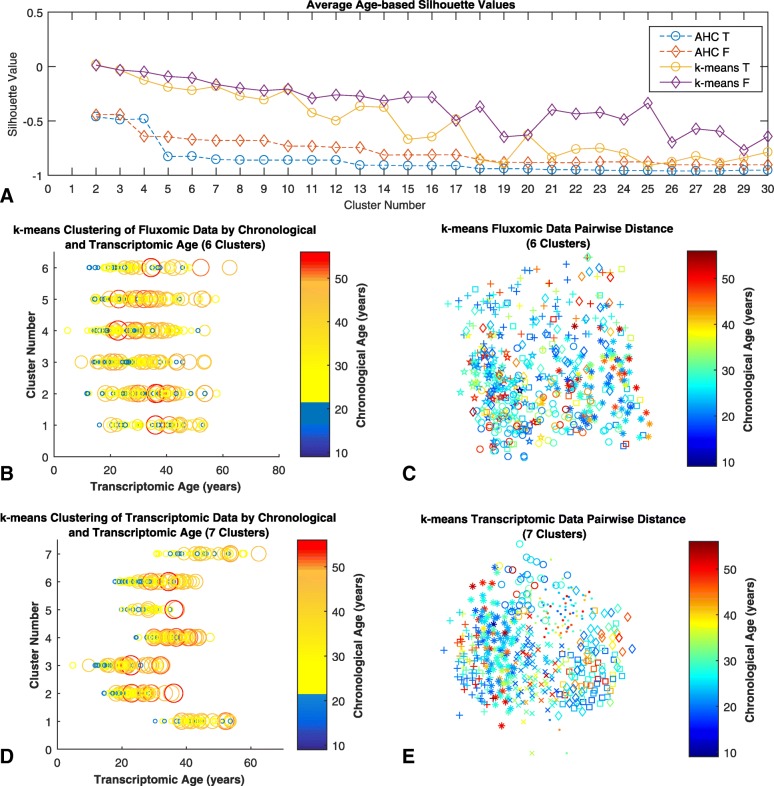
k-means clustering with age. We propose and investigate an average age-based silhouette value **a**. This is calculated using the chronological age, and clustering data from both hierarchical and k-means clustering. The silhouette values are calculated by averaging all the individuals’ silhouette scores, for a number of clusters ranging from 2 to 30. k-means clustering performs consistently better than hierarchical clustering. In both types of clustering, fluxomic data clusters better with chronological age than transcriptomic data. The pairwise distance of clusters with chronological age is visualised in scatter plots for both transcriptomic **e** and fluxomic **c** data. Clusters are annotated with different shapes, while age is shown with colour. Individual clusters are plotted against transcriptomic and chronological age for both transcriptomic **d** and fluxomic **b** data. Note that since transcriptomic age was calculated from transcriptomic data, we would expect to see more distinction in transcriptomic age between clusters for the transcriptomic data

From the plot of average age-based silhouette values (Fig. 2a), optimal cluster numbers were chosen from the point closest to 1 at which there is an ’elbow bend’ in the curve, indicating a drop in the amount of variance explained by the clusters after this point [60]. For the transcriptomic data seven clusters were chosen, while for fluxomic data six clusters were chosen. The pairwise distance of the chosen cluster numbers and the results of clustering for both transcriptomic and fluxomic data with chronological and transcriptomic age are shown in Figs. 2e, 2c, 2d, and 2b respectively. We selected k-means as a clustering algorithm because it performed consistently better than hierarchical clustering both in the transcriptomic (Kolmogorov-Smirnov test statistic =0.66, *p*-value =2.91·10^−6^) and in the fluxomic-based clustering (Kolmogorov-Smirnov test statistic = 0.8, *p*-value =5.59·10^−9^).

Remarkably, with both methods, and considering a variable number of clusters between 2 and 30 (Fig. 2a and Additional file 2), fluxomic-based clustering consistently outperformed transcriptomic-based clustering in terms of age-based average silhouette values (Kolmogorov-Smirnov test statistic =0.38, *p*-value =0.022 for k-means; Kolmogorov-Smirnov test statistic =0.62, *p*-value =1.15·10^−5^ for hierarchical). We also analysed the pattern of silhouette values based on deviations from linearity. In general, we found that drops in silhouette values corresponded to smaller clusters being merged into much larger, less distinct clusters, or cluster boundaries changing such that there was a loss of intra-cluster cohesion and inter-cluster separation (more details can be found in Additional file 3). Our results therefore suggest that, compared to gene expression values, individual-based poly-omic models and their predicted flux rates are a better predictor of chronological age.

### Principal component analysis identifies predictors of ageing

The number of components to retain in the analysis was determined by their eigenvalues and the total contribution to variance explained by the components. Three criteria were applied to the data: (i) according to Kaiser’s criterion [61] only those components with eigenvalus greater than 1 should be retained; (ii) the overall contribution to variance of the retained components should be 50% or greater; and (iii) for an *n*×*m* matrix, if the data were randomly distributed the expected contribution to variance of the eigenvalue for each axis would be 100/(*n*−1) % in terms of rows [62]. Therefore, any axis with a contribution to variance larger than this proportion should be retained as “significant”. The threshold percentage variable contributions to variance for each group used in the analysis is shown in Table 1. The resulting number of components retained for analysis for each group is shown in Table 2.

**Table 1:** Eigenvalue threshold % variance values for PCA

Group	Individuals	Average	Threshold % Variance
All individuals	499	100/498	0.2
21-	112	100/111	0.9
22-49	360	100/359	0.28
50 +	27	100/26	3.85

Significant components had eigenvalues with an individual contribution to variance greater than the threshold percentage variance value. The threshold percentage variances for each of the different groups of data are shown along with the details of how that value was obtained

**Table 2:** Number of components retained in PCA

Group	Components Retained	% Variance
All individuals	151	74.88
21-	39	63.42
22-49	112	71.79
50 +	10	55.14

For each group, the number of significant components are shown along with their cumulative contribution to variance

Using only the significant components, the overall contribution of each reaction to component variability was calculated using (5) for each of the three age groups, 21- (21 and under), 22-49 and 50 + (50 and over). The contributions of each of the reactions to the different age groups were then compared by calculating the difference between them. The differences between the reaction contributions for (i) 21- and 22-49, (ii) 21- and 50 +, and (iii) 22-49 and 50 + were obtained in order to determine the reactions that vary the most with age (see Additional file 4).

In the results of the analysis of the differences in overall contribution of the 95 pathways, four pathways appeared in the top 20 of all the age group comparisons: CoA synthesis, vitamin D metabolism, hyaluronan metabolism and pyruvate metabolism. All four of these pathways also decreased in their contribution with age. Pyruvate and CoA are both key components of the citric acid cycle, which is essential in energy production in the mitochondria. Reduced stamina observed in the ageing population is thought to be related to impairment of mitochondrial energy production [63, 64]. Hypovitaminosis D in the ageing population is a major cause of impaired bone formation and mineralisation (osteoporosis) [65, 66]. Hyaluronan or Hyaluronic acid (HA) has a high capacity to bind and retain water molecules and is found in high levels in the extracellular matrix of skin where it regulates skin moisture. Reduced levels of HA are associated with the loss of moisture in ageing skin [67, 68]. Changes in HA size contribute to age-related impairment of wound healing in skin [69, 70] and to the viscoelasticity of synovial fluid, which can contribute to osteoarthritis, a common disease of ageing [71–73].

In the 21- to 22-49 group, the overall difference in contribution values for all but one of the top 20 pathways increased from the 21- group to the 22-49 group. Interestingly, CoA synthesis increased but CoA catabolism decreased, suggesting higher CoA levels due to increased synthesis and decreased degradation. Conversely, for the 21- group and 50 + group, the overall contribution values for all but one of the top 20 pathways decreased. Only Vitamin B2 metabolism increased. This is consistent with recent studies showing that the activity of vitamin B2 metabolism does not decrease until after the age of 50 [74]. All of the top 20 pathways contribution values decreased for the 50 + group compared to the 22-49 group.

Squalene and cholesterol synthesis, vitamin A metabolism and glycine, serine, alanine and threonine metabolism overall contributions all decrease with age. Sebum, produced by the sebaceous glands, contains both cholesterol and squalene. Squalene is correlated with *α*-tocopherol (vitamin E) levels on the surface of the skin. *α*-tocopherol is the main antioxidant on the skin [75] and its decrease with ageing may contribute towards the signs of ageing skin [76, 77].

The reactions that make up the squalene and cholesterol synthesis pathway are part of the mevalonate pathway, which produces the precursors of all the steroid hormones, heme cholesterol, coenzyme Q_10_, and vitamin K [78]. The decrease in plasma levels of high density lipoprotein (HDL) cholesterol is correlated with a higher risk of atherosclerosis [79]. Low vitamin K has been associated with osteoarthritis and impaired cognitive function in older adults [80, 81]. The synthesis of mitochondrial coenzyme Q_10_ can decrease with age [82]; this constitutes an important factor for health, as coenzyme Q_10_ is an antioxidant that protects against diseases that involve oxidative stress, such as cardiovascular and neurogenerative diseases [83–88]. Furthermore, the synthesis of heme, the major functional form of iron in the body, decreases with age [89, 90] and has been linked with neurodegenerative disorders such as Alzheimer’s disease [91]. Steroid hormones are also known to decline with age [92, 93]; decreasing sex steroid hormone deficiency in oestrogens and androgens contributes to ageing skin [94, 95] and increased risk of cardiovascular disease in women [93, 96].

Glycine, serine, alanine and threonine are all non-essential amino acids. Decreasing glycine levels have been linked with age-associated oxidative stress [97], while age-associated decrease in serine metabolism has been linked with impaired memory function in the brain [98, 99]. Alanine metabolism is associated with the liver, and alanine transaminase has been suggested as a biomarker for ageing [100]. Vitamin A cannot be produced by the body and is therefore obtained through diet; a decrease in the active form of vitamin A in the body, retinol, is thought to be linked with age-associated slowing in visual dark-adaption [101, 102].

**Fig. 3:**
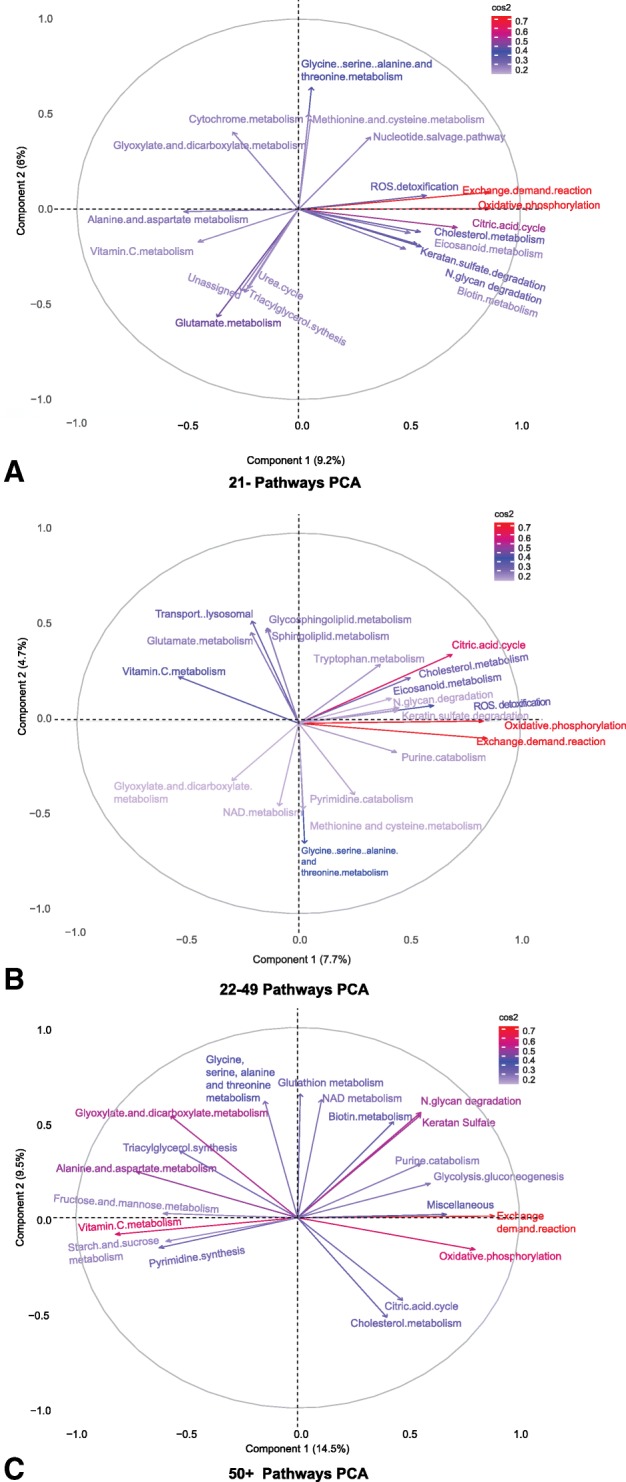
**Principal component analysis factor maps.** Factor maps of the top 20 contributing pathways for components 1 and 2 in each age group, 21-, 22-49 and 50 + (**a**, **b**, **c**). The quality of the contribution of each pathway is shown by its colour

Figure 3a-c show the factor maps for the top 20 pathways contributing to variance for each age group. For the 21- group, the overall contribution of components 1 and 2 to variation is 15.2*%*. For the 22-49 group, it is 12.4*%* and for the 50 + group it is 24*%*. If the variance was evenly distributed across all pathways, then the expected average variation would be 100/95 *%*=1.05*%*. Interestingly, the variance explained by the first two components in the 50+ group is higher compared to the other two groups, suggesting that less latent pathways, but with an increasingly strong role, characterise the ageing phenotype.

**Fig. 4:**
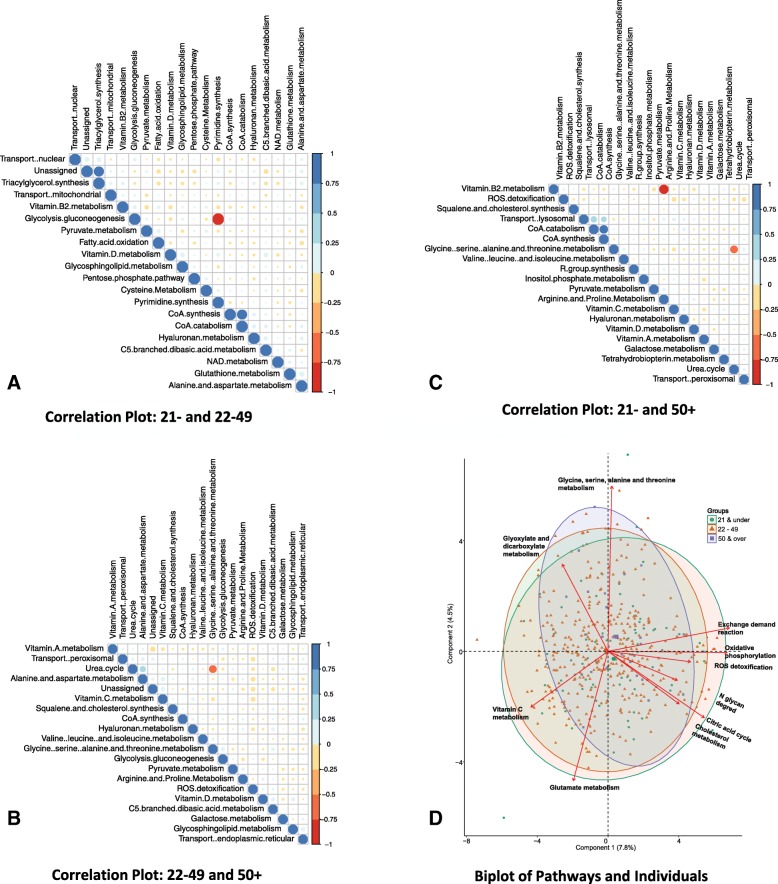
**Principal component analysis applied to pathways.** The correlation plots of fluxes are shown for the pathways with the top-20 overall difference in each group, 21- and 22-49, 21- and 50 +, and 22-49 and 50 + (**a**, **b**, **c**). The plots show the large amount of intercorrelation between pathways. A biplot of individuals grouped by age and the top 10 pathways contributing to overall variance of components 1 and 2 is also shown **d**. We note some differentiation between the three age groups, with the biggest differentiation shown in the axis of the 50 + group compared to the other two. The 50 + group appears to lie along the glyoxylate and dicarboxylate metabolism axis

As a result, the biplot (Fig. 4d) of individuals grouped by age and the top 10 pathways contributing to overall variance of components 1 and 2 shows differentiation between the three age groups, with the biggest differentiation shown in the axis of the 50 + group compared to the other two. The 50 + age group appears to lie along the glyoxylate and dicarboxylate metabolism axis. To identify the amount of intercorrelation in the pathway flux data, correlation plots were created for the pathways with the top 20 overall difference in contribution in each group, 21- and 22-49, 21- and 50 +, and 22-49 and 50 + (Fig. 4a-c). The plots show the large amount of intercorrelation between pathways.

Exchange/demand reaction and oxidative phosphorylation remain the high contributors to variance for all three age groups. The most notable rise in contribution to variation with age is vitamin C metabolism, which moves from rank 18 in the 21- to rank 2 in the 50 + group. Changes in oxidative stress are known to affect the levels of vitamin C in the body [103]. Furthermore, oxidative damage to the genome caused by ROS (reactive oxygen species) is thought to be one of the causes of ageing [104]. Interestingly, ROS detoxification is one of the top 10 contributors in the 21- and 22-49 groups but does not appear in the top 20 contributors in the 50 + group, suggesting a possible link with ageing, oxidative stress and the changing variation contributed by vitamin C metabolism. Glyoxylate and dicarboxylate metabolism also has a large rise in contribution to variance with age. Glyoxylate and dicarboxylate metabolism is strictly linked with glycine, serine and threonine metabolism, pyruvate metabolism, ascorbate metabolism (a mineral salt of vitamin C), all of which our results have identified as linked to ageing.

### Elastic net regression identifies metabolic-age predictors

The analysis was performed using tenfold cross validation for both metabolic reactions and metabolic pathways. The results of the metabolic pathways analysis are reported here, while the results for the metabolic reactions can be found in Additional file 1. Elastic net regression of the 95 metabolic pathways returned 100 possible *λ* values with their associated effect sizes. The value *λ*=1.57 was chosen as this had the lowest mean squared error value. This gave metabolic effect sizes for three pathways: butanoate metabolism, pyrimidine synthesis and beta-alanine metabolism (Table 3).

**Table 3:** Metabolic effect sizes

Subsystem	Effect size
Butanoate metabolism	-0.004462535
Pyrimidine synthesis	-2.18609E-05
beta-Alanine metabolism	-0.002443117

The effect sizes on age of the three metabolic pathways selected by elastic net regression

Both butanoate and beta-alanine metabolism have been linked to sarcopenia, deterioration of skeletal muscle, with age [105]. Butanoate and beta-alanine metabolism are facilitated by the enzyme aldehyde dehydrogenase. One of the substrates of this enzyme is nicotinamide adenine dinucleotide (NAD). Reversal of NAD loss as we age is currently undergoing human trials following successful age reversal of mitochondrial function in the skeletal muscle cells of mice [106]. Mitochondrial dysfunction, which is linked to ageing, also leads to a reduction of pyrimidine synthesis [107, 108].

In order to calculate the biological/metabolic ages of the individuals, equations (1) and (2) were modified to use the metabolic effect sizes. We therefore define the following metabolic predictor:

9 $$ M = \sum\limits_{i} b_{i} f_{i},   $$

where *M* is the metabolic predictor, *f*_*i*_ is the flux value of the *i*th pathway, and *b*_*i*_ is the effect size for the *i*th pathway. The *metabolic age* was then defined as:

10 $$ \ SM = \mu_{age} + \left(M-\mu_{M}\right) \frac{\sigma_{age}}{\sigma_{M}},   $$

where *μ*_*age*_ and *σ*_*age*_ are the mean and the standard deviation of the chronological age, while *μ*_*M*_ and *σ*_*M*_ are the mean and the standard deviation of the metabolic predictor *M*.

The metabolic ages showed correlation (*p*-value =4.7·10^−4^) with chronological age. Both the metabolic ages and the chronological ages of all 499 individuals can be found in Additional file 1, which also includes the results of the regression performed directly on reactions. This is a promising starting point for a metabolic age predictor, which can be further refined using more samples to improve predictor accuracy.

## Conclusion

While chronological age gives an accurate measurement of the time since an individual’s birth, biological age – also called transcriptomic age – gives a more accurate representation of the relative health of an individual compared to others of the same age. Where an individual has a biological age greater than their chronological age, they are ageing more quickly than their peers, and therefore have decreased life expectancy. Finding predictors of biological ageing and measuring their presentation in individuals can allow targeted and personalised interventions, both medication and lifestyle-based, to improve health and life expectancy.

A number of age-associated genes along with their effect sizes have previously been identified in the literature and used to calculate the biological age of individuals [33]. The limitation of this approach is that it does not take into account the metabolic effects of those gene expression values on the cellular phenotype. For example, a gene that has been found to be differentially expressed with age may have little or no effect on cellular metabolism. Here we have achieved the first steps towards a metabolic age predictor to overcome the limitations of previous transcriptomic-only approaches.

Using a genome-scale metabolic model of CD4 T-cells combined with transcriptomic data, we were able to obtain individual-specific metabolic models and generate fluxomic data (Additional file 5). The subsequent stage of our analysis used a novel application of the silhouette method and clustering techniques, from which we identified that fluxomic data clusters better with chronological age, therefore suggesting that metabolic models are a better predictor of the chronological age of an individual. Applying PCA analysis and elastic net regression enabled us to identify potential metabolic predictors of ageing. Many of these predictors have also been identified in the literature as linked to the ageing process, validating the reliability of the method. Finally, elastic net regression produced metabolic age predictors and their effect sizes, from which we calculated the metabolic age of each individual.

Our next step will be to further refine the metabolic age predictors obtained from elastic net regression with more data from individuals across the different age ranges. Future studies could also use other age predictors, such as epigenetic clocks [109] and telomere length [110] as variables to investigate how they correlate with our metabolic age predictor. Furthermore, although proteomics data has some limitations, including providing less coverage than transcriptomic data [111, 112], it can be included as a complementary method to improve the accuracy of the results. We will also investigate whether there are differences in the metabolic age predictors based on different phenotypes/attributes of individuals such as gender. This will allow further refinement of predictors based on individual data, and will suggest more personalised interventions to reduce metabolic age and improve life expectancy.

## Additional files

Additional file 1 Metabolic ages (XLSX 33 kb)

Additional file 2 Silhouette values raw data (XLSX 11 kb)

Additional file 3 Plots of k-means clusters (PDF 283 kb)

Additional file 4 Supplementary PCA analysis raw data (XLSX 412 kb)

Additional file 5 Fluxomic data (XLSX 9174 kb)
