# Supplementary material for: The poly-omics of ageing through individual-based metabolic modelling
Source: BMC Bioinformatics. 2018 Nov 20;19(Suppl 14):415. doi: 10.1186/s12859-018-2383-z (PMC6245500; doi:10.1186/s12859-018-2383-z)
Supplement: Supplementary file 3 — Plots of k-means clusters (PDF 283 kb) [file 12859_2018_2383_MOESM3_ESM.pdf]

Plots of k-means Clusters

This file contains plots of clustered data used for comparison to analyse the cause of drops in the silhouette values. In the fluxomic k-means data we analysed the drop in silhouette values between clusters 16 and 17 and between clusters 18 and 19. We found that the cohesion of the clusters represented by the numbers 5, 9 and 12 in the plot of 16 clusters was lost in the plot of 17 clusters . Similarly, the cohesion of the clusters represented by the numbers 5, 6, 15 and 17 in the plot of 17 clusters plot were lost in the plot of 18 clusters.

In the transcriptomic k-means data we analysed the drop in silhouette values between clusters 10 and 11, 14 and 15 and 17 and 18. In the plot of 10 clusters the clusters represented by the numbers 2 and 4 lose their distinction in the plot of 11 clusters as they are merged into one large cluster. Similarly the clusters represented by the numbers 10 and 11 in the plot of 14 clusters are merged into a less distinct single cluster in the plot of 15 clusters. Additionally, the clusters represented by the numbers 2, 9 show better cohesion in the plot of 14 clusters. In the comparison of the plots of 17 and 18 clusters the cluster represented by the number 17 is particularly better defined in the plot of 17. Clusters 7, 15 and 16 are also better defined in the plot of 17 clusters.

The plots of the clusters used for comparison can be found below.

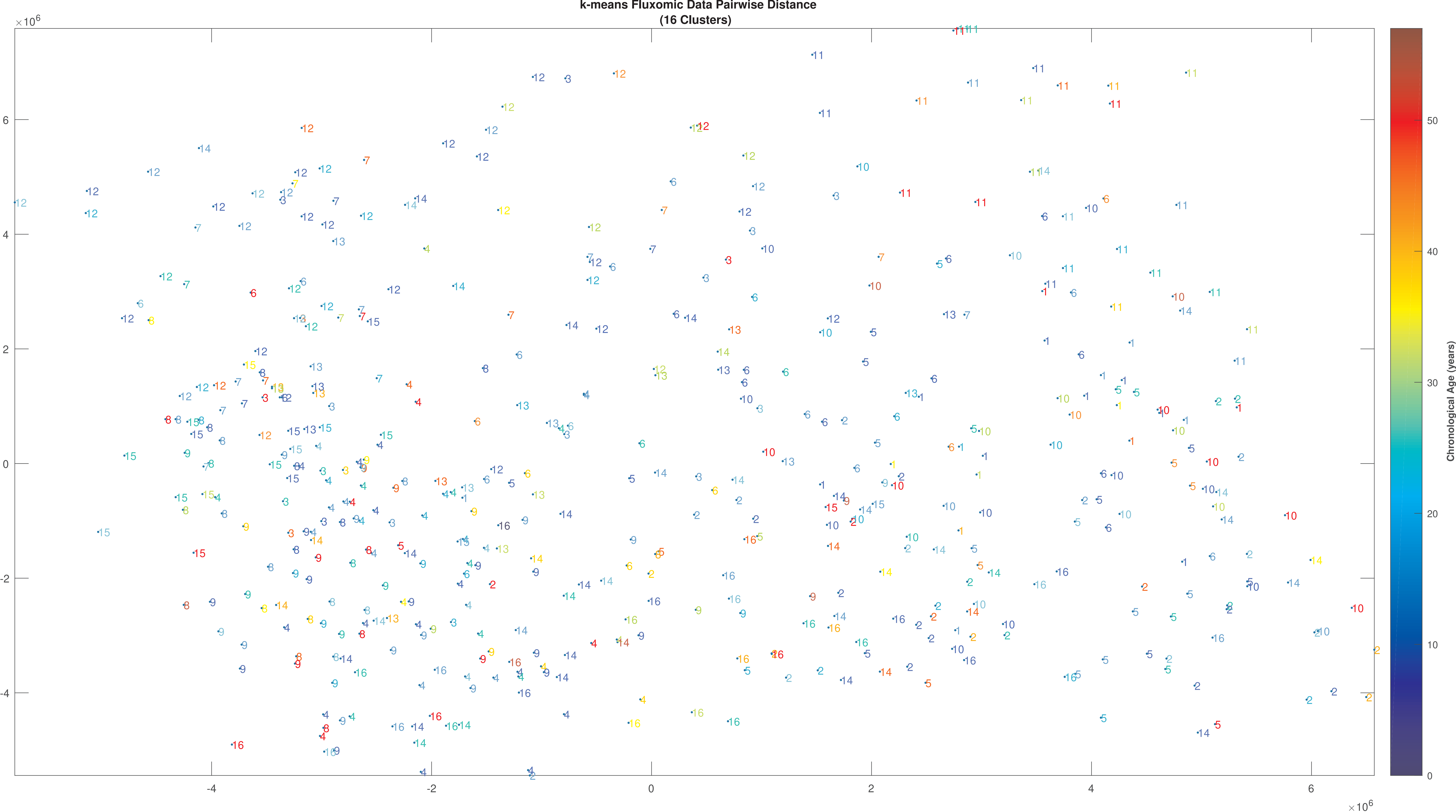
k-means Fluxomic Data Pairwise Distance
(16 Clusters)
×10^6
Chronological Age (years)

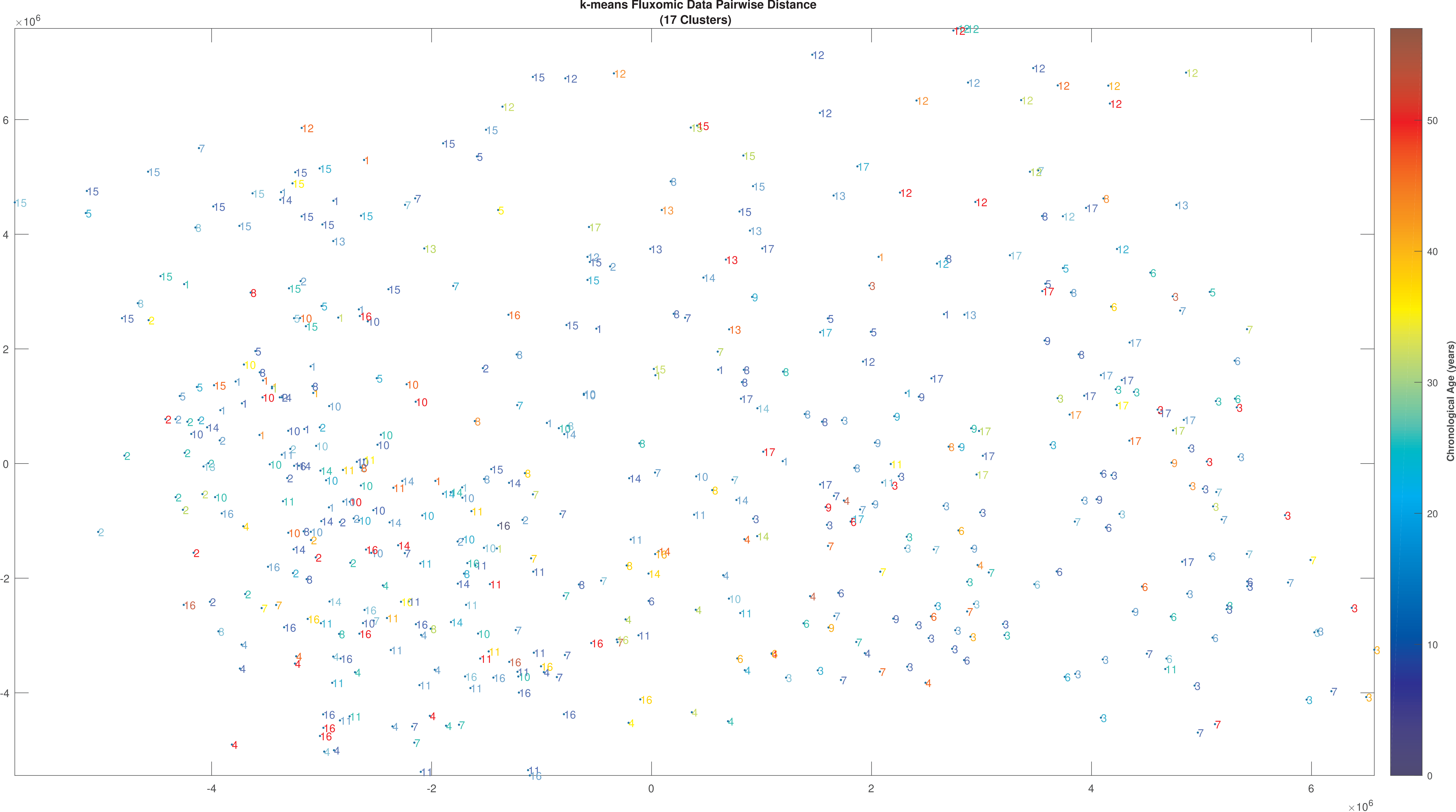

k-means Fluxomic Data Pairwise Distance
(17 Clusters)
×10^6
×10^6
Chronological Age (years)

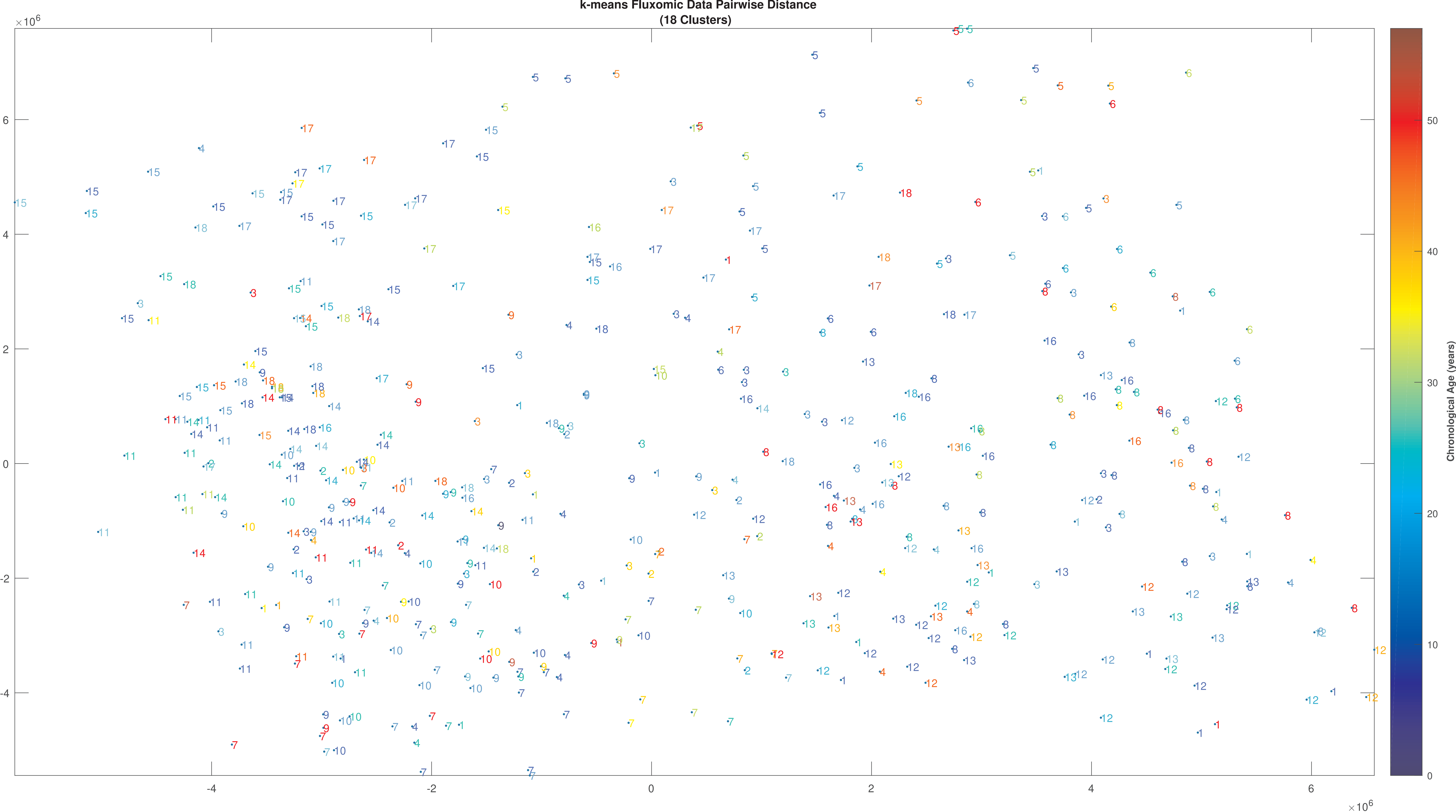
k-means Fluxomic Data Pairwise Distance
(18 Clusters)
×10^6
×10^6
Chronological Age (years)

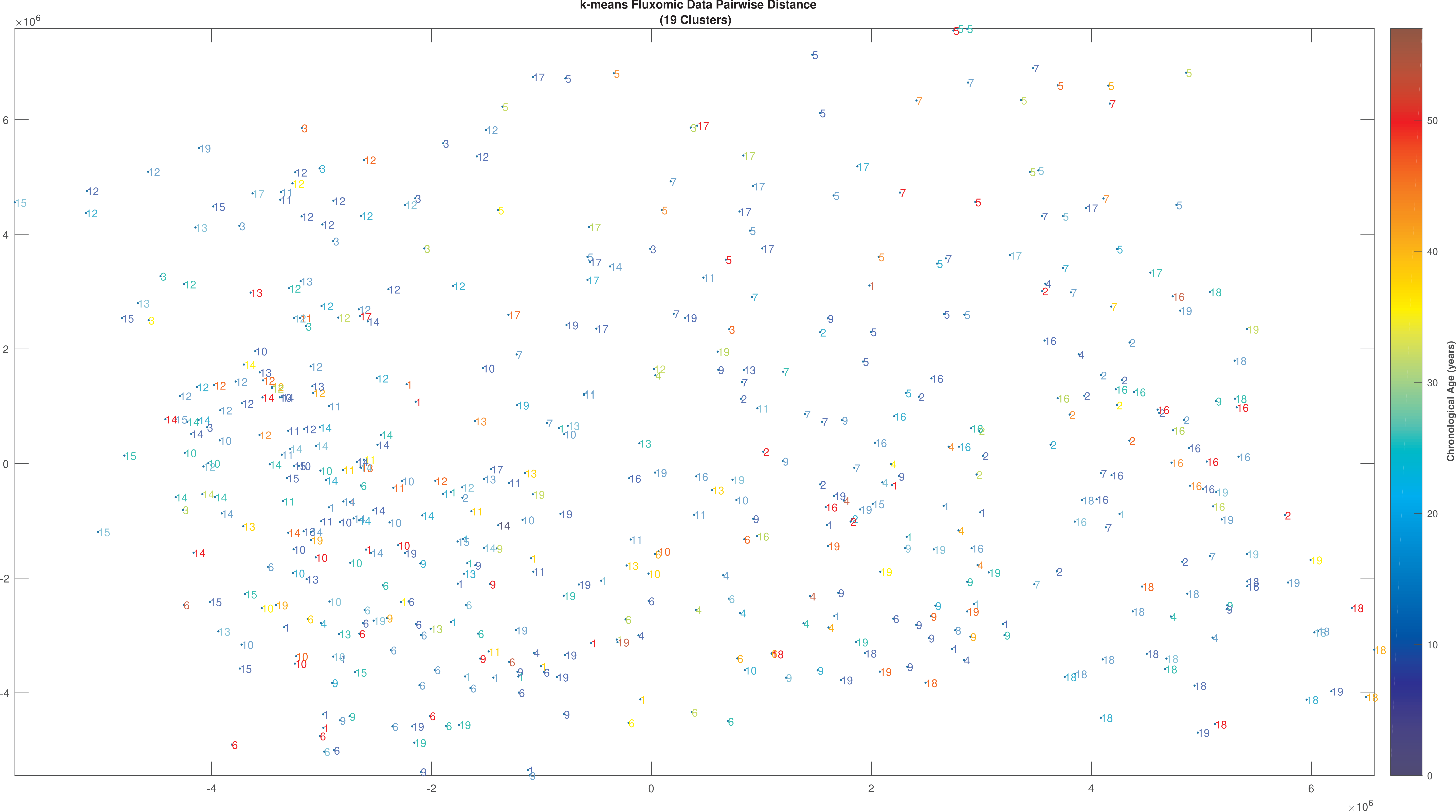
k-means Fluxomic Data Pairwise Distance
(19 Clusters)
×10^6
6
4
2
0
-2
-4
-4
-2
0
2
4
6
×10^6
Chronological Age (years)
0
10
20
30
40
50

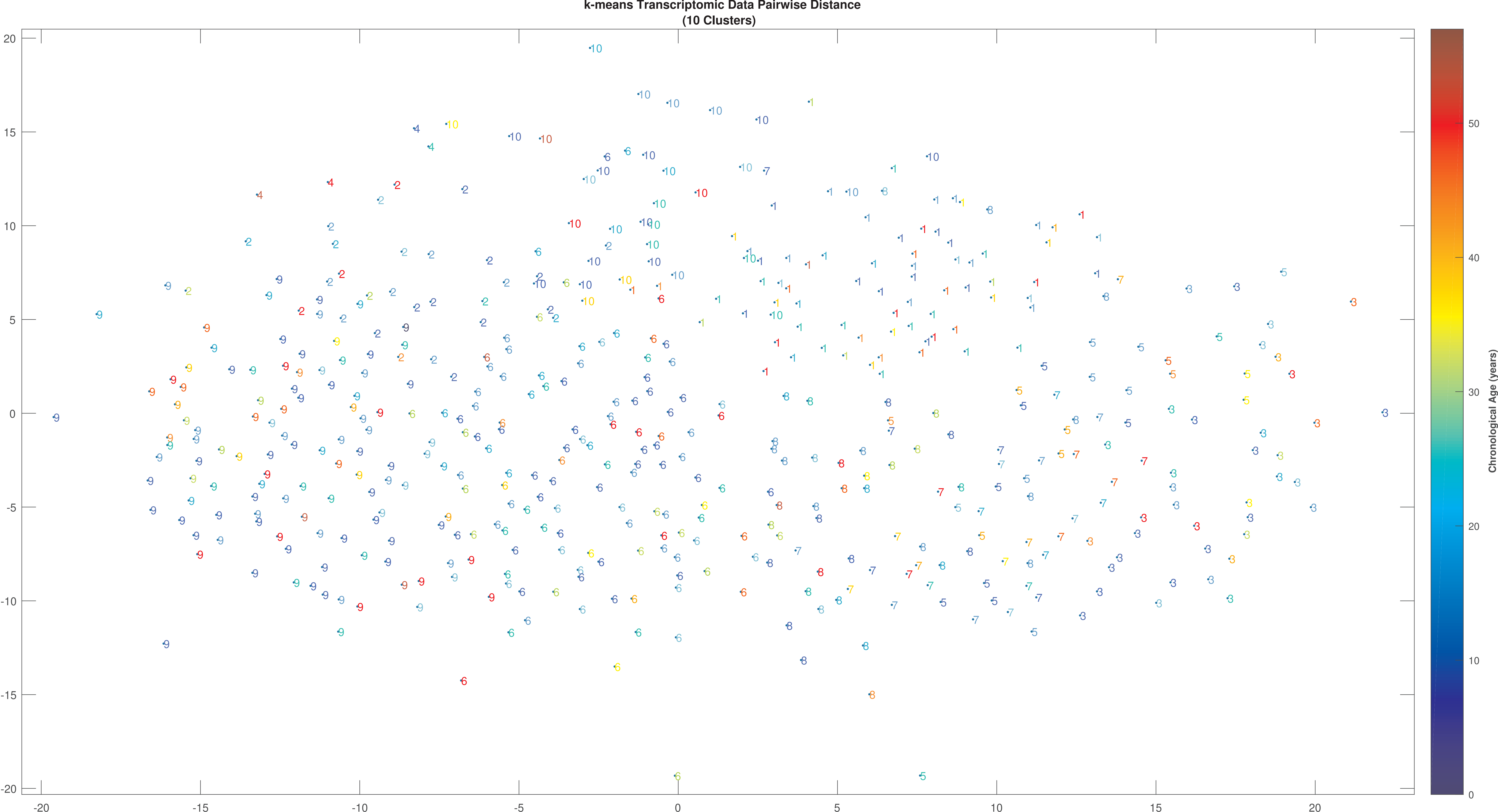
k-means Transcriptomic Data Pairwise Distance
(10 Clusters)
Chronological Age (years)

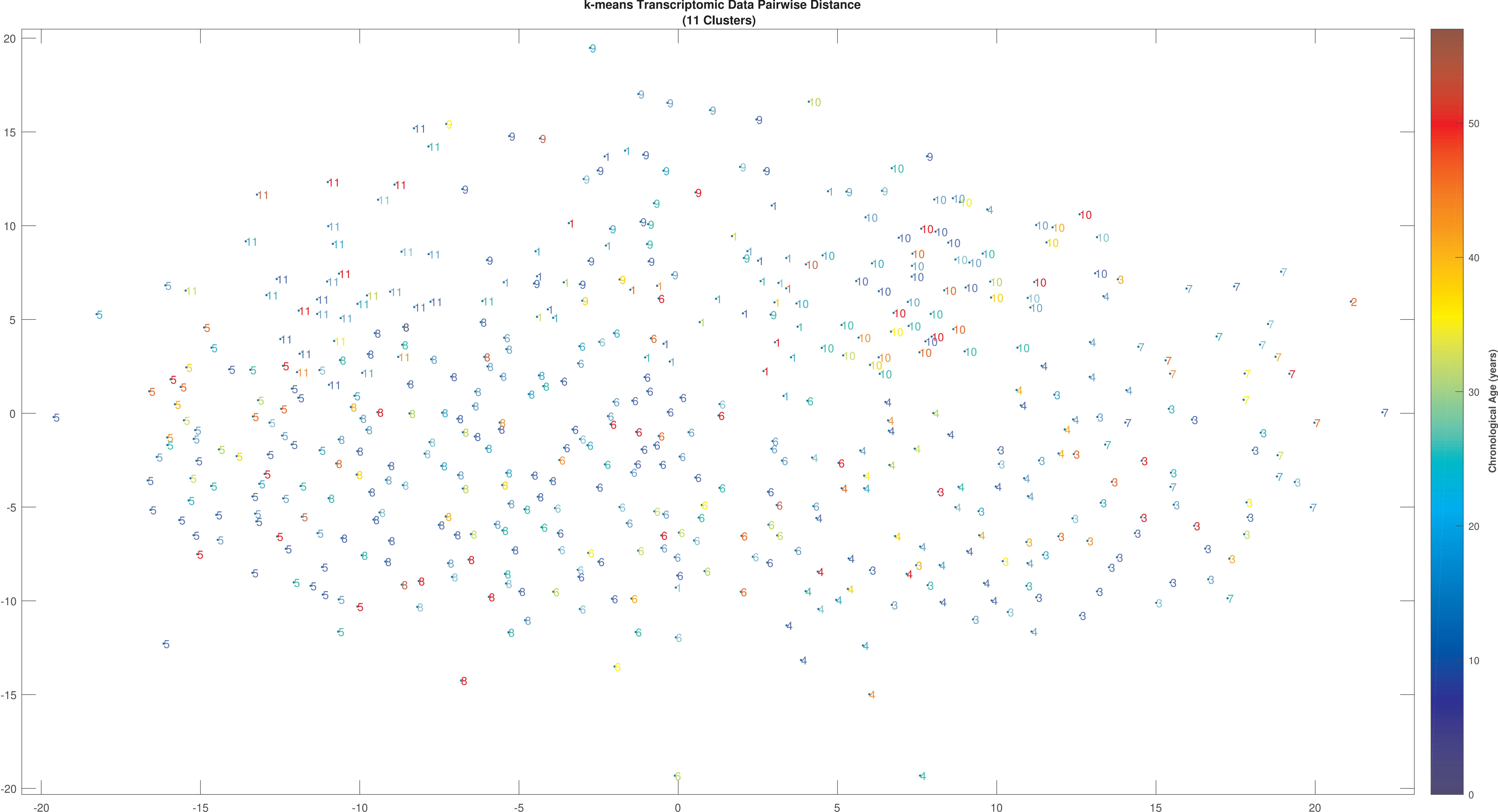
k-means Transcriptomic Data Pairwise Distance
(11 Clusters)
Chronological Age (years)

k-means Transcriptomic Data Pairwise Distance
(14 Clusters)

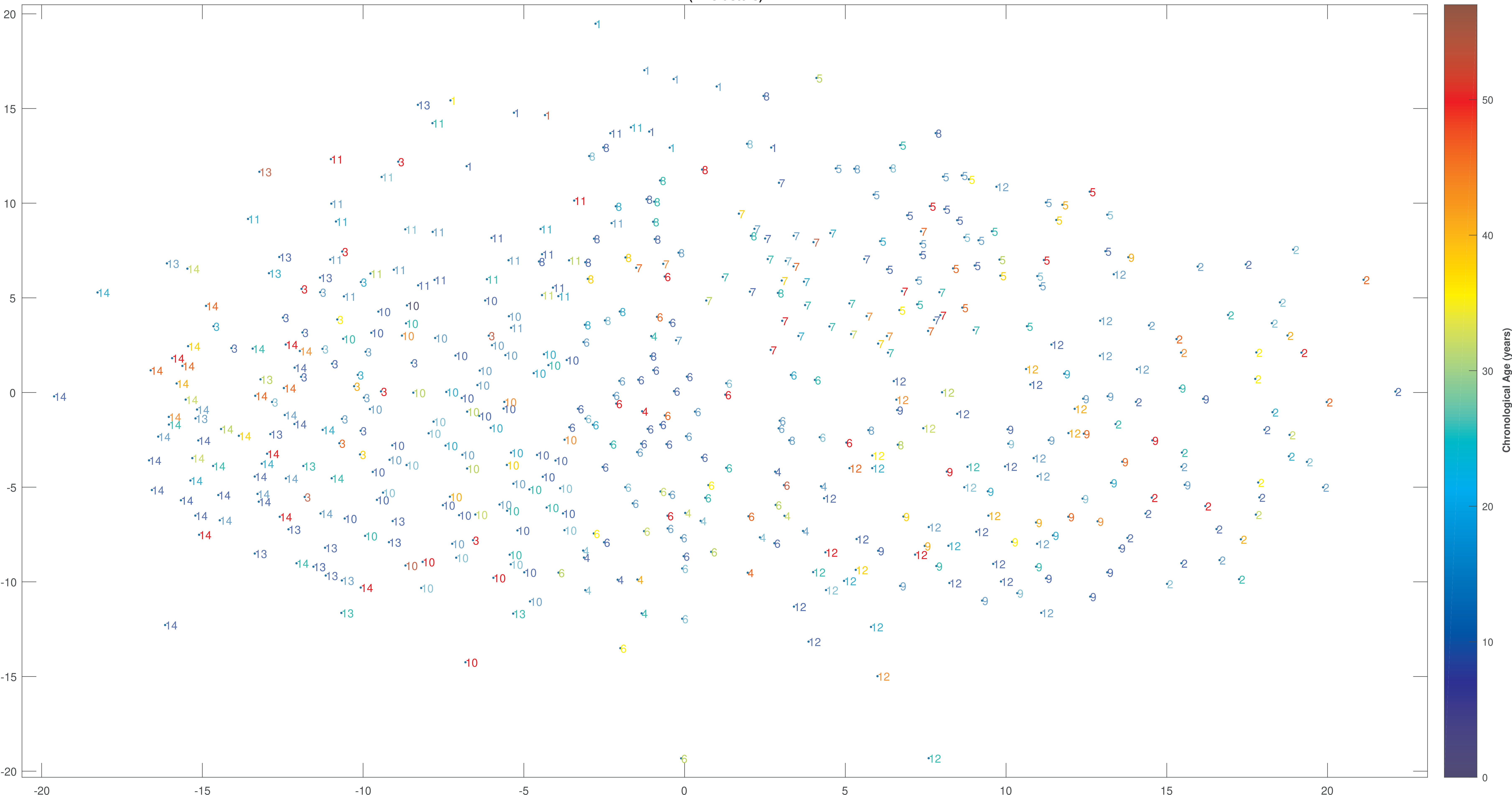

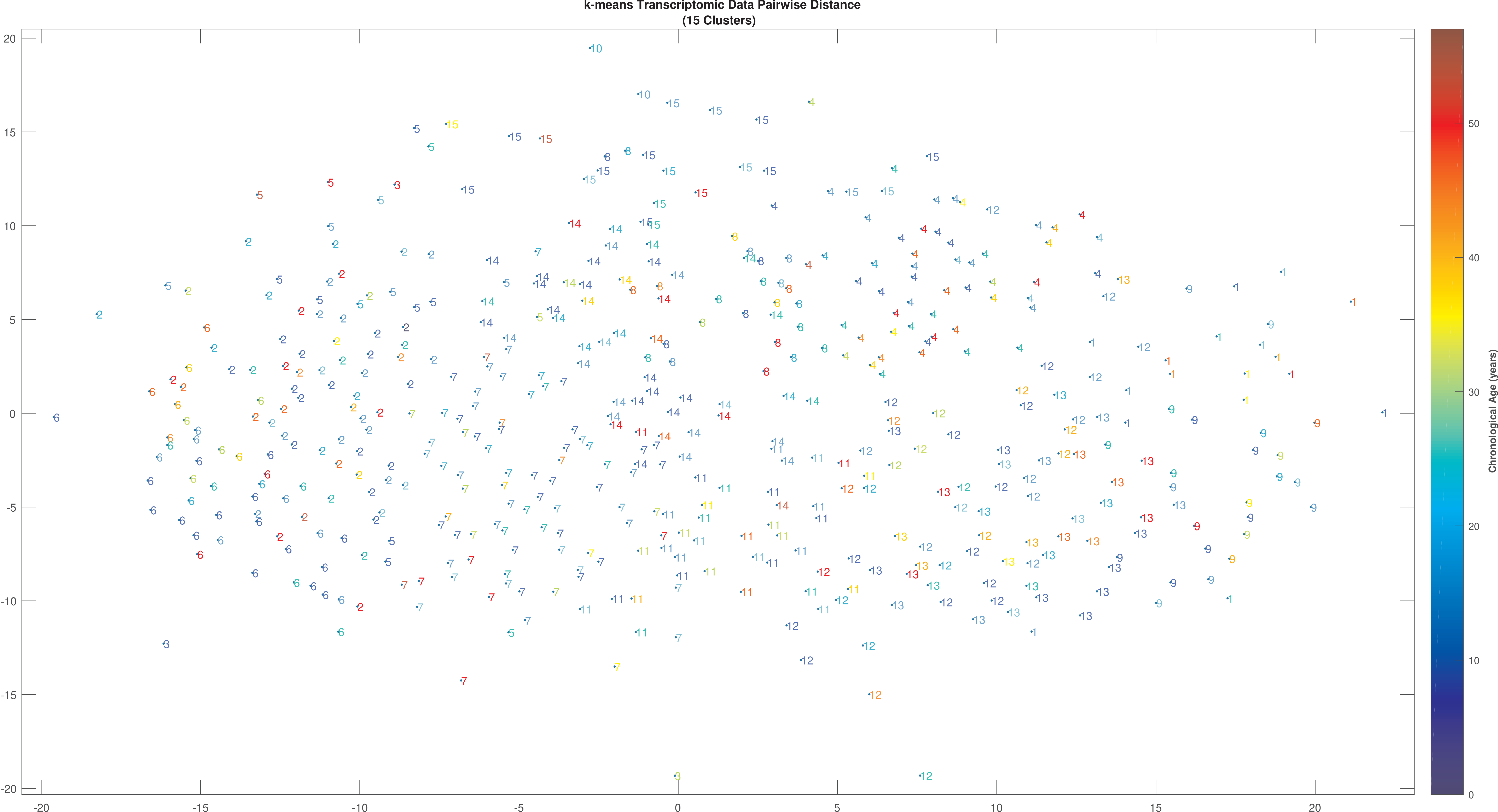
k-means Transcriptomic Data Pairwise Distance
(15 Clusters)
Chronological Age (years)
0
10
20
30
40
50
-20
-15
-10
-5
0
5
10
15
20

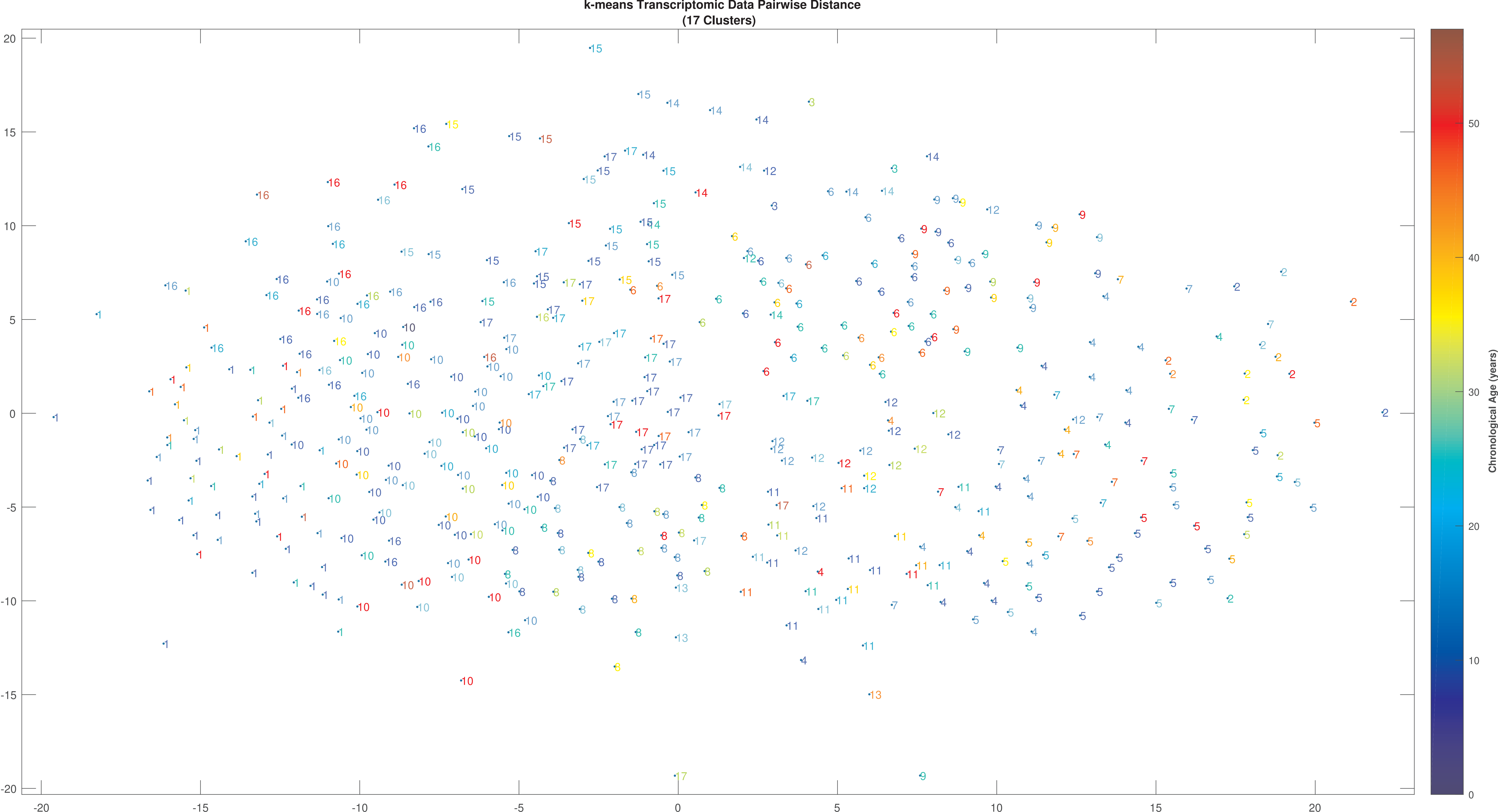
k-means Transcriptomic Data Pairwise Distance
(17 Clusters)
Chronological Age (years)

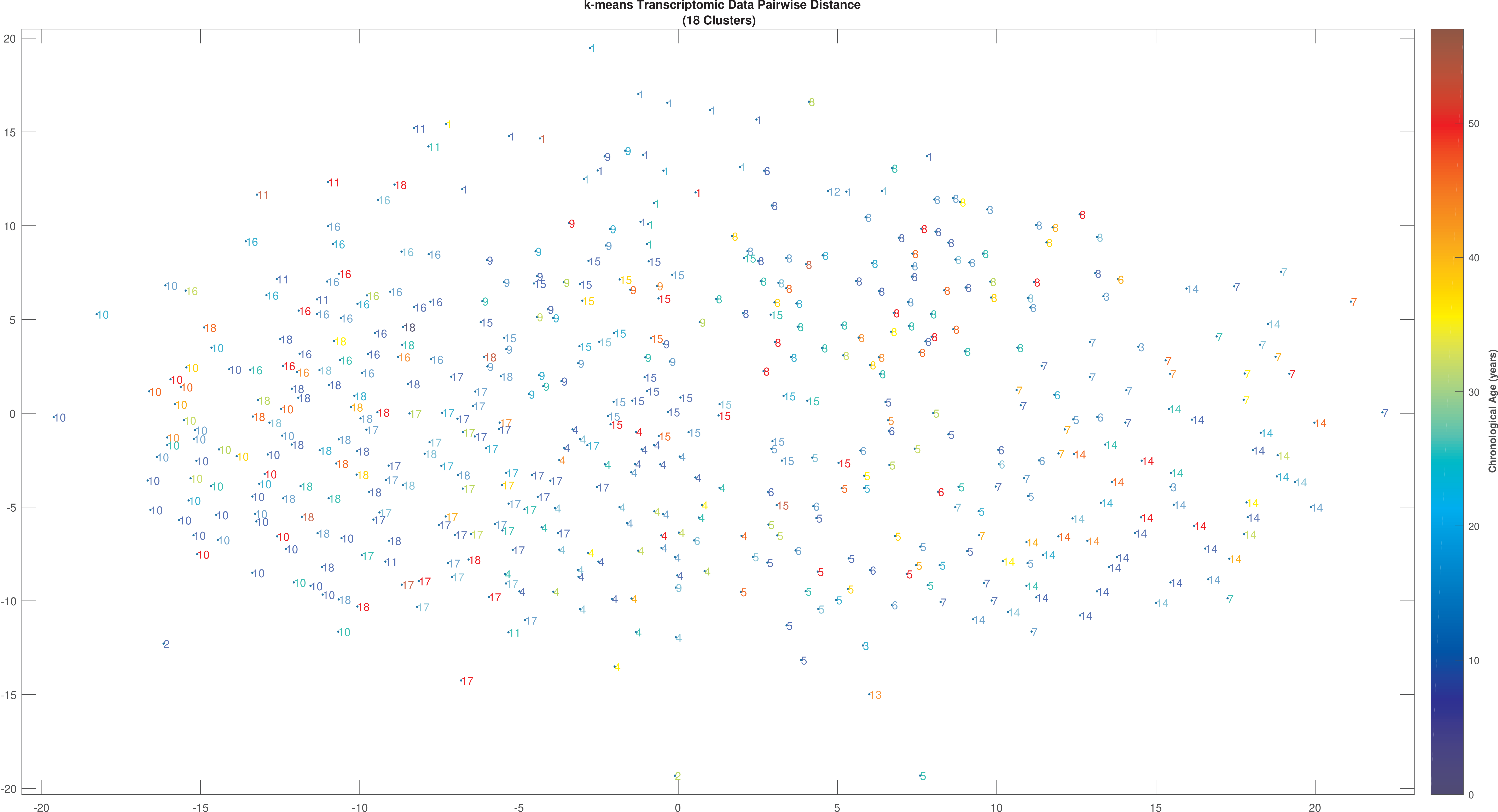
k-means Transcriptomic Data Pairwise Distance
(18 Clusters)
Chronological Age (years)
-20
-15
-10
-5
0
5
10
15
20
